# Enhanced Notch Activation Is Advantageous but Not Essential for T Cell Lymphomagenesis in Id1 Transgenic Mice

**DOI:** 10.1371/journal.pone.0032944

**Published:** 2012-02-29

**Authors:** Hong-Cheng Wang, Vincent Peng, Ying Zhao, Xiao-Hong Sun

**Affiliations:** 1 Immunobiology and Cancer Research Program, Oklahoma Medical Research Foundation, Oklahoma City, Oklahoma, United States of America; 2 Oklahoma School of Science and Mathematics, Oklahoma City, Oklahoma, United States of America; Garvan Institute of Medical Research, Australia

## Abstract

T cell lymphoblastic leukemia (T-ALL) is known to be associated with chromosomal abnormalities that lead to aberrant expression of a number of transcription factors such as TAL1, which dimerizes with basic helix-loop-helix (bHLH) E proteins and inhibits their function. Activated Notch receptors also efficiently induce T cell leukemogenesis in mouse models. Interestingly, gain-of-function mutations or cryptic transcription initiation of the Notch1 gene have been frequently found in both human and mouse T-ALL. However, the correlations between these alterations and overall Notch activities or leukemogenesis have not been thoroughly evaluated. Therefore, we made use of our collection of T cell lymphomas developed in transgenic mice expressing Id1, which like TAL1, inhibits E protein function. By comparing expression levels of Notch target genes in Id1-expressing tumors to those in tumors induced by a constitutively active form of Notch1, N1C, we were able to assess the overall activities of Notch pathways and conclude that the majority of Id1-expressing tumors had elevated Notch function to a varying degree. However, 26% of the Id1-expressing tumors had no evidence of enhanced Notch activation, but that did not delay the onset of tumorigenesis. Furthermore, we examined the genetic or epigenetic alterations thought to contribute to ligand-independent activation or protein stabilization of Notch1 and found that some of the Id1-expressing tumors acquired these changes, but they are not uniformly associated with elevated Notch activities in Id1 tumor samples. In contrast, N1C-expressing tumors do not harbor any PEST domain mutations nor exhibit intragenic transcription initiation. Taken together, it appears that Notch activation provides Id1-expressing tumor cells with selective advantages in growth and survival. However, this may not be absolutely essential for lymphomagenesis in Id1 transgenic mice and additional factors could also cooperate with Id1 to induce T cell lymphoma. Therefore, a broad approach is necessary in designing T-ALL therapy.

## Introduction

T cell lymphoblastic leukemia (T-ALL) is often associated with chromosomal translocations and alterations that lead to dysregulation of the expression of a range of transcription factors or alteration of their functions [1], [2]. For example, aberrant expression of the TAL1 gene is found in more than half of the childhood T-ALL cases, which is frequently due to the t (1;14) translocation or intergenic deletions upstream of the TAL1 gene [1], [2]. TAL1 is a basic helix-loop-helix (bHLH) protein and dimerizes with bHLH E proteins (represented by E2A) to bind to DNA [3]. While complexes containing TAL1, E2A, LMO2, GATA1 and LDB1 are capable of activating transcription of genes involved in erythroid differentiation [4], heterodimers of TAL1 and E2A exhibit diminished transcriptional activity in comparison to E2A homodimers [5], [6]. This raises the possibility that TAL1, when expressed out of its natural contexts, acts as a dominant-negative inhibitor to predispose T cells to leukemogenesis [7]–[9].

This notion is supported by observations that ablation of the E2A gene leads to the formation of T cell lymphoma in mice [10], [11]. Consistently, the oncogenic potential of TAL1 has been shown to be independent of its transcriptional and DNA binding activity but requires the HLH dimerization domain [8]. Furthermore, we and others have shown that expression of the Id proteins diminishes the DNA binding activity of E proteins, blocks T cell development and causes T cell lymphomas in transgenic mice at high frequencies and penetrance [12]–[15]. Therefore, E proteins have been regarded as tumor suppressors, at least in the T lymphoid lineage.

Constitutive activation of Notch receptors have also been shown to have potent oncogenic effects on T cells, inducing T cell leukemia in less than two months [16], [17]. Unlike the TAL1 gene, chromosomal translocation events that cause Notch activation are rarely found in human T-ALL patients [18]. However, gain-of-function point mutations of the Notch1 gene have been found in a large fraction of human T-ALL [19]–[21]. Likewise, mutations have also been detected in T cell lymphomas developed in mouse models lacking the function of transcription factors such as Ikaros and E2A [15], [22]–[24]. Point mutations in the heterodimerization (HD) domain and the PEST sequence of the Notch1 gene render the protein to be spontaneously activated and resistant to proteasome-mediated degradation, respectively [19]. The HD domain mutations have been shown to possess more potent transforming activity than mutations in the PEST sequence [25]. While the mutations in the HD domain are commonly found in human T-ALL, these alterations are relative rare in mouse T-ALL [21]. Instead, several mechanisms involving cryptic initiation of Notch1 transcription have recently been shown to produce truncated Notch1 proteins. They include alternative promoter usage resulted from 5′ deletion or RAG-mediated recombination and intragenic initiation of Notch1 transcription at regions between exons 25 and 29 [26]–[28]. These alterations could have similar effects as the HD domain mutations found in human T-ALL and result in the ligand-independent activation of Notch1.

Given the potent oncogenic effects of Notch signaling in T-ALL animal models, spotlights have been focused on Notch when considering therapeutic strategies. Inhibitors of γ-secretases have been shown to elicit cell cycle exit in T-ALL cells and induce apoptosis [29]–[31]. However, a clinical trial with this family of inhibitors showed no objective clinical responses and due to the intestinal toxicity of Notch inhibition, the trial was terminated [32]. Furthermore, the majority of the data from the studies correlating the incidence of Notch mutations and clinical outcomes of T-ALL indicate that activating mutations of the Notch1 gene or inactivating mutations of the *Fbxw7* gene, which encodes a ubiquitin ligase instrumental for Notch degradation, are associated with a favorable prognosis to conventional therapies [33]–[36]. These observations highlight the need for a better understanding of the contribution of Notch activation in the pathogenesis of T-ALL.

Since Notch activation is found to overlap with other genetic alterations in most of the T-ALL cases [1], it is important to ask whether Notch signaling is the critical initiating factor of T cell leukemogenesis while abnormalities of various transcription factors enable Notch activation. Conversely, it is possible that gain-of-function mutations of the Notch signaling pathway accumulates under selective pressure for the survival and proliferation of leukemic cells which harbor other oncogenic factors or lack certain tumor suppressors. It is also important to note that despite the observations of these genetic or epigenetic alterations of the Notch1 gene, their correlations with Notch activities in leukemic cells remain to be carefully determined.

We are particularly interested in examining the relationship between Notch activation and loss of E2A function. Although E proteins have been shown to be involved in the transcriptional activation of the Notch1 gene [37], the situations in T cell lymphomas resulted from E protein deficiency may be entirely different. Previous data on gain-of-function Notch mutations have been mostly obtained using E2A deficient T-ALL cell lines and in TAL1 transgenic tumors [24], [38], [39]. Here, we made use of our collections of T cell lymphomas developed in mice expressing Id1 or Notch1 intracellular domain (N1C). By comparing levels of best-known Notch target genes and incidences of gain-of-function events in Id1 and N1C-expressing tumor samples, we conclude that a large fraction of Id1-expressing tumors exhibit evidence of significant Notch activation. However, a substantial fraction of tumors do not show elevated expression of Notch target genes, suggesting that Notch signaling is dispensable for T cell lymphomagenesis. Furthermore, intragenic transcription initiation occurs in Id1 but not N1C-expressing tumors, and it correlates with increased Notch activities to some extent. Yet, exceptions have been found in several cases where cryptic starts of transcription do not result in Notch activation. Findings from these studies suggest that although multiple genetic and epigenetic alterations that lead to Notch activation accumulate through selective pressure for growth or survival of Id1-expressing lymphoma cells, tumorigenesis can occur independently of Notch activities. Therefore, simple Notch targeting therapy might not be sufficient for eradicating T-ALL.

## Results

### Activation of Notch signaling pathways in Id1 transgenic T cell lymphomas

**Figure 1:**
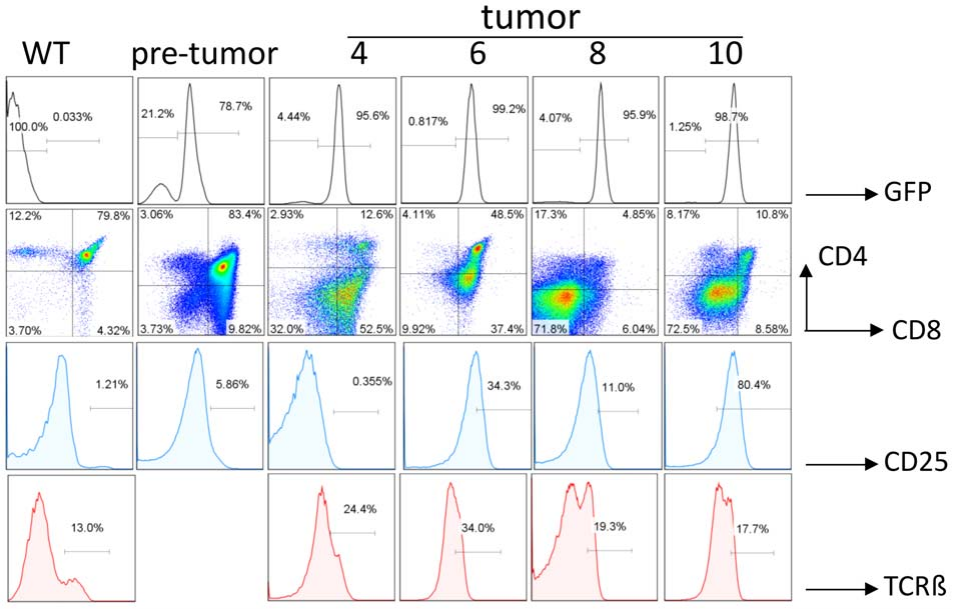
Characterization of N1C-expressing T cell lymphoma. Single cell suspensions from normal and tumor tissues were analyzed using FACS. The percentage of GFP was determined after excluding PI positive cells and by using wild type thymocytes as a negative control. Thymocytes from a 22 day-old ROSA26-stop-N1C/lck-Cre mouse were used as a pre-tumor control. Percentages of cells of indicated surface phenotypes are label within the plots. The designations of the tumor samples on top of the plots correspond to the identifications of these tumors used throughout the study.

**Figure 2:**
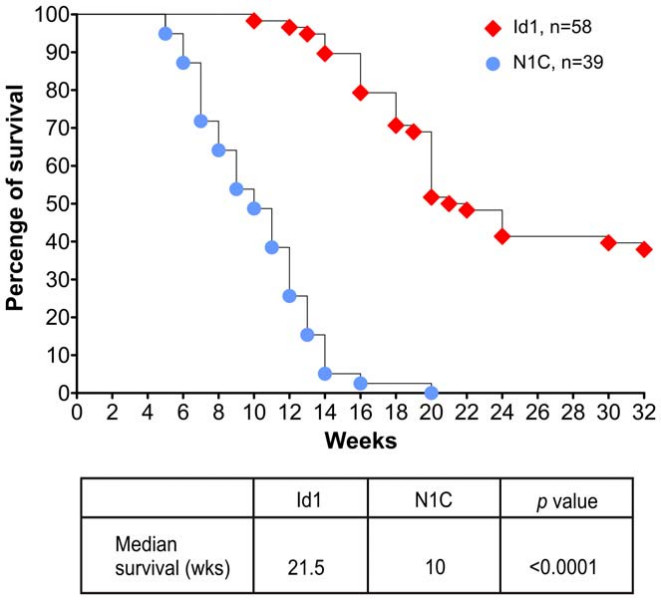
Survival curves of Id1 and N1C-expressing mice. Id1 transgenic mice and ROSA26-stop-N1C/lck-Cre mice were monitored for signs to tumor growth judging by hunched back and labored breathing. Median survival and statistics were determined using the Prism software and Log-rank (Mantel-Cox) Test.

We have previously shown that transgenic expression of Id1 under the control of the lck proximal promoter leads to the formation of T cell lymphoma in the thymus with high penetrance and a median survival of 21.5 weeks [15]. We have archived a series of these tumor samples and analyzed the CD4 and CD8 expression profile of each tumor. Since the tumors are usually 10–50 times the size of a normal thymus, the majority of the cells collected are likely derived from tumor cells. FACS analyses usually showed a predominant population of cells (data not shown). In addition, we also have a collection of tumors harvested from ROSA26-Stop-N1C mice crossed with lck-Cre transgenic mice. Like the Id1 tumors, NIC-expressing tumors are also large in size and almost invariably metastasize to lymph nodes, liver or/and kidney. Representative FACS profiles of these tumors are shown in Fig. 1. NIC-expressing tumors exhibit different surface phenotypes with regard to CD4, CD8, CD25 and TCRβ expression. Since expression of N1C is turned on by Cre recombinase driven by the lck promoter, which was also used to control Id1 expression in the transgenic mice, we were interested in comparing the tumorigenic potential between Id1 and N1C. In contrast to the median survival of 21.5 weeks determined for Id1 mice, that of N1C-expressing mice was 10 weeks, indicating a much more potent oncogenic effect of N1C than Id1 (Fig. 2).

**Figure 3:**
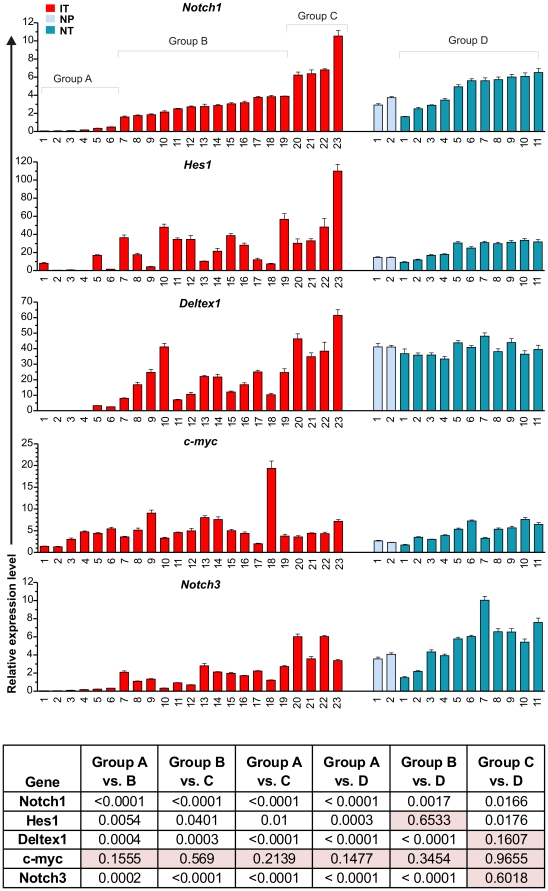
Quantitative analyses of expression of Notch target genes in Id1 and N1C-expressing tumors and normal tissues. Total RNA was isolated from archived Id1 and N1C tumor samples or fresh thymocytes. Real-time PCR analyses were performed with primers for indicated genes. Levels of transcripts were normalized against that of β-actin by calculating ΔC_T_. Expression levels relative to that of a control generated from cDNA of wild type thymocytes were determined by using the 2^−ΔΔCT^ formula. Data shows the average with standard deviations obtained from triplicates, which was calculated as described [50]. Throughout the manuscript, Id1-expressing tumors are designated as “IT” (red) whereas N1C-expressing pre-tumor and tumor samples are called “NP” (light blue) and “NT” (blue), respectively. Statistical analyses were performed using Student's *t* tests among groups A to D as marked on top of the first bar graph. *p* values are presented in a table shown at the bottom. Values above 0.05 are highlighted in light pink.

To compare the extent of Notch activation in Id1 and N1C-expressing tumors, we randomly selected a set of samples of each genotype and examined expression levels of Notch1 as well as representative Notch downstream target genes including Hes1, Deltex1, c-myc and Notch3 [40]. The 23 Id1-expressing tumors are designated as IT1 to 23, while the 11 N1C-expressing tumors are dubbed NT1 to 11. Two thymus samples without any obvious signs of tumor growth from 3-week old N1C-expressing mice were also included as pre-tumor controls, called NP1 and NP2. Real-time PCR analyses were performed and levels of expression were normalized to a reference control generated from total wild type thymocytes so that the relative levels of each transcript expressed in all samples are comparable. In Fig. 3, data from Id1 and N1C-expressing tumors are arranged in ascending orders with regard to endogenous Notch1 expression detected by PCR amplification with primers binding to the 3′ region of the transcript, and the samples are named accordingly.

**Figure 4 pone-0032944-g004:**
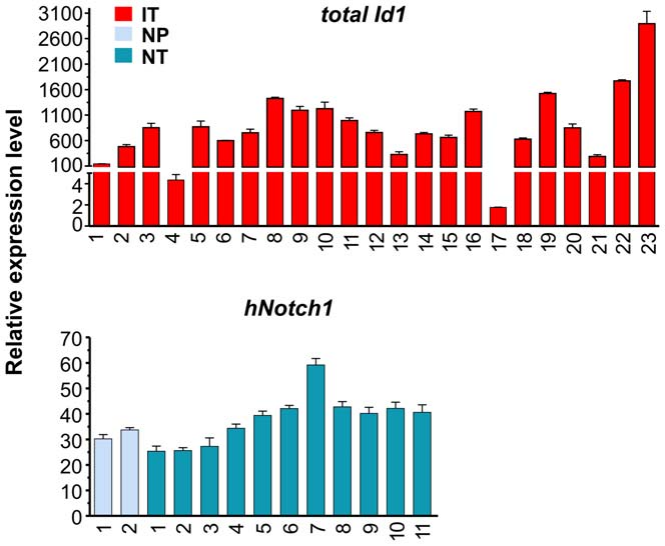
Expression of the transgenes. Levels of Id1 and human N1C transcripts were determined as described for Figure 3.

Expression of human N1C in pre-tumor thymocytes from the ROSA26 locus activated transcription of its known downstream targets such as endogenous Notch1, Hes1, Deltex1, c-myc and Notch3 (Fig. 3). Consistently, N1C-expressing tumors showed uniformly high levels of expression of Notch targets, proportional to the levels of human N1C (Fig. 4). One exception is the uncharacteristically lower level of c-myc expression in NT7 despite higher levels of Notch1 and 3 in this sample.

Among the Id1-expressing tumors, the 23 samples can be divided into three groups, groups A to C, based on Notch1 expression (Fig. 3). Group C includes sample IT20-23, which expressed comparable or higher levels of endogenous Notch1 and Hes1 relative to N1C-expressing tumors (Group D). Their levels of Deltex1, c-myc and Notch3 were similar to those of N1C tumors. In contrast, Group B (IT7-19) produced significantly lower levels of Notch1, Deltex1 and Notch3 compared to Group C and D (*p* values are less than 0.002 from the Student's t test) whereas the difference for Hes1 levels was less remarkable. There are also inconsistencies among different Notch targets. For example, IT9 had a low level of Hes1 but high levels of Deltex1 and c-myc. On the other hand, IT18 generated extremely high levels of c-myc whereas those of Hes1, Deltex1 and Notch3 were rather low compared to other samples in this group. Nonetheless, Groups B and C, which constitutes 74% of the samples analyzed, exhibited evidence of elevated Notch signaling as indicated by the expression of various Notch target genes.

However, Group A, which represents the remainder 26% of the samples (IT1-6), showed extremely low levels of Notch1, Deltex1 and Nocth3 expression. Hes1 levels were also very low except for two samples, IT1 and 5 (Fig. 3). Thus, it appears that Notch activity is minimal in these 6 tumors. Surprisingly, c-myc levels in these tumors were comparable to samples in other groups. In fact, no statistical significance was found in the differences of c-myc expression among any of the groups, thus raising the question if the c-myc gene is activated independently of Notch signaling (Fig. 3).

As controls, we measured levels of transgene expression in both Id1 and N1C tumors. Very high levels of Id1 expression were detected in all but 2 tumors whereas N1C expression was relatively uniform in all tumors (Fig. 4).

### Intragenic transcription initiation of the Notch1 gene contributes to Notch activation in Id1 tumors

**Figure 5 pone-0032944-g005:**
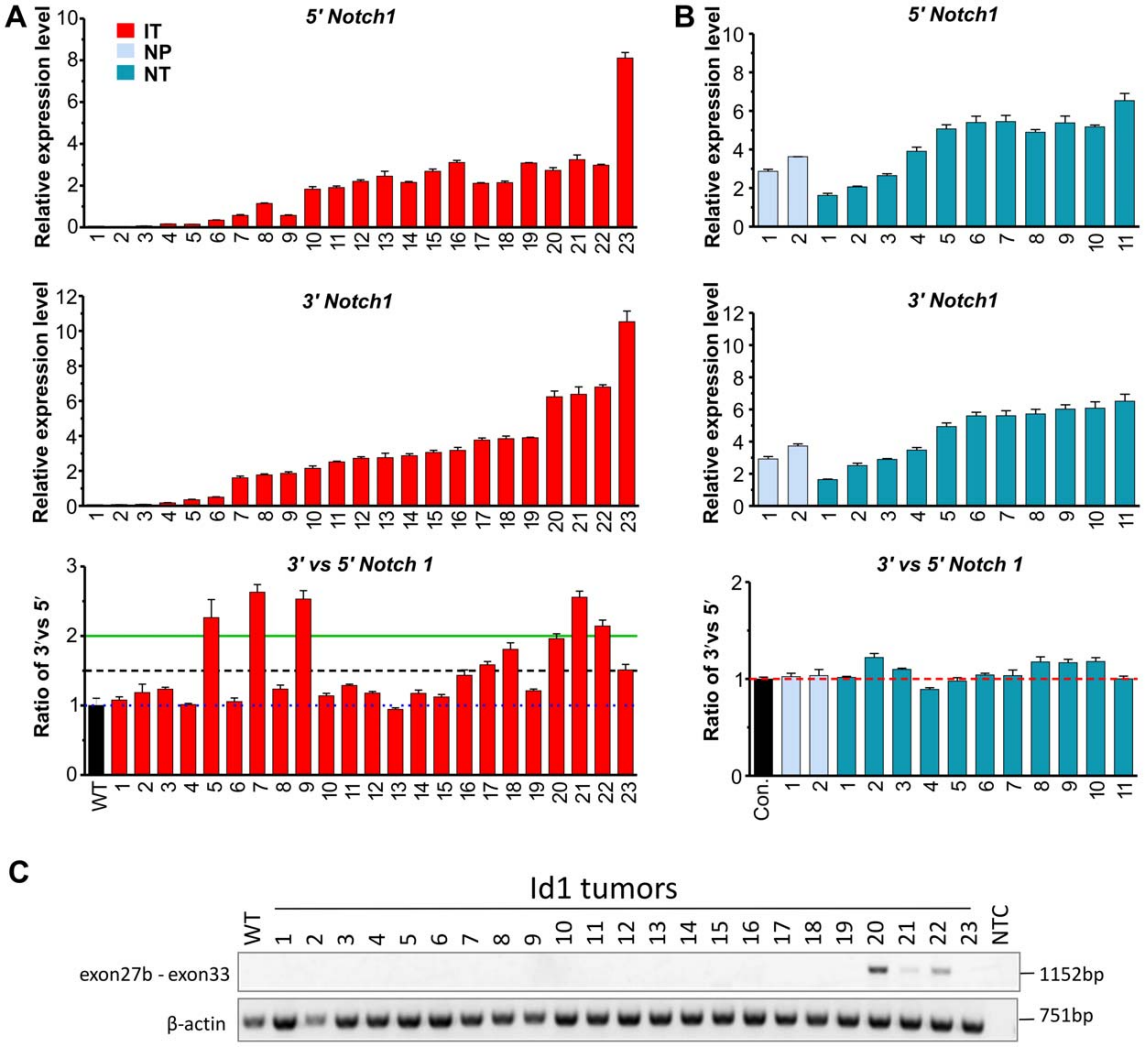
Examination of intragenic transcription initiation of the Notch1 gene. Real-time PCR analyses were carried out using Id1 (A) and N1C (B) samples described in Fig. 3. Primer pairs amplifying the 5′ and 3′ regions of Notch1 bind to sequences encoded by exons 5 to 6 and 33 to 34, respectively. Data for 3′ Notch1 is the same as those shown in Fig. 3. The ratio of levels of 3′ to 5′ Notch1 in each sample was compared to the average levels in wild type mice obtained from 6 FVB/N (A) and 4 C57BL/6 (B) mice, respectively. (C) PCR amplification using primers binding to intron 27 and exon 33 for an intragenic transcript designated as exon 27b-exon 33. Amplification of β-actin serves as a quality control of cDNA. Products were analyzed on agarose gel electrophoresis and inversed photograph is shown. NTC, no template control.

To understand how Notch activities are elevated in these tumor cells, we examined several mechanisms known to aberrantly activate Notch signaling. First, it has recently been reported that intragenic transcription initiation occurs in the region spanning exon 25 to exon 29 of the Notch1 gene as a result of the loss of Ikaros function or 5′-end deletion of the Notch1 gene [26]–[28]. The resulting cryptic transcripts encode truncated Notch1 receptors that are capable of ligand-independent proteolytic cleavages, generating intracellular domains of Notch1 capable of transcriptionally activating its downstream targets. To test if these events occurred in Id1 transgenic T cell lymphomas, we determined levels of Notch1 transcripts by using PCR primers specific for exons 5–6 and exons 33–34 to amplify Notch1 transcripts via the 5′ and 3′ ends, respectively (Fig. 5A). The levels of the 5′ and 3′ transcripts in total thymocytes of 6 individual wild type mice were also measured. As reported previously [26]–[28], wild type thymocytes only transcribe full-length Notch1 mRNA, and therefore the average ratio of the level of 3′ transcripts over that of 5′ transcript in wild type thymocytes was set as 1. Subsequently, the ratio in each tumor sample was determined and normalized against the wild type value. This result has been verified by using additional pairs of PCR primers binding to 5′ and 3′ ends of the transcript, respectively (data not shown). We considered a ratio of 1.5 to be highly significant based on the assumption that one of the two Notch1 alleles produces the cryptic transcripts at the same level as the full-length species. By this criterion, 9 out of the 23 tumors appeared to generate the truncated message and hence the activated form of Notch1 protein. In contrast, T cell lymphomas developed in N1C-expressing mice did not show any sign of cryptic intragenic transcription initiation by the same measurements (Fig. 5B).

To further validate this finding, we PCR-amplified an intragenic transcript initiated in intron 27 by using a specific 5′ primer that does not recognize conventional transcripts [26]. As shown in Fig. 5C, no product was obtained using cDNA of wild type thymocytes as a template. However, a product of expected size was detected in IT20-22 but not in other IT samples or NT samples (Fig. 5C, data not shown). This product was sequenced and found to match the sequence between intron 27 and exon 33. Therefore, it appears that 3 of the 9 samples thought to harbor intragenic transcripts based on the 3′ to 5′ ratio actually initiated transcription from a cryptic promoter in intron 27. Other samples with high 3′ to 5′ ratios might have started transcription in other locations between exons 25 and 29, for which PCR detection methods are currently not available.

### Alternative promoter usage at the 5′ end of the Notch1 gene is activated by Notch signaling

**Figure 6:**
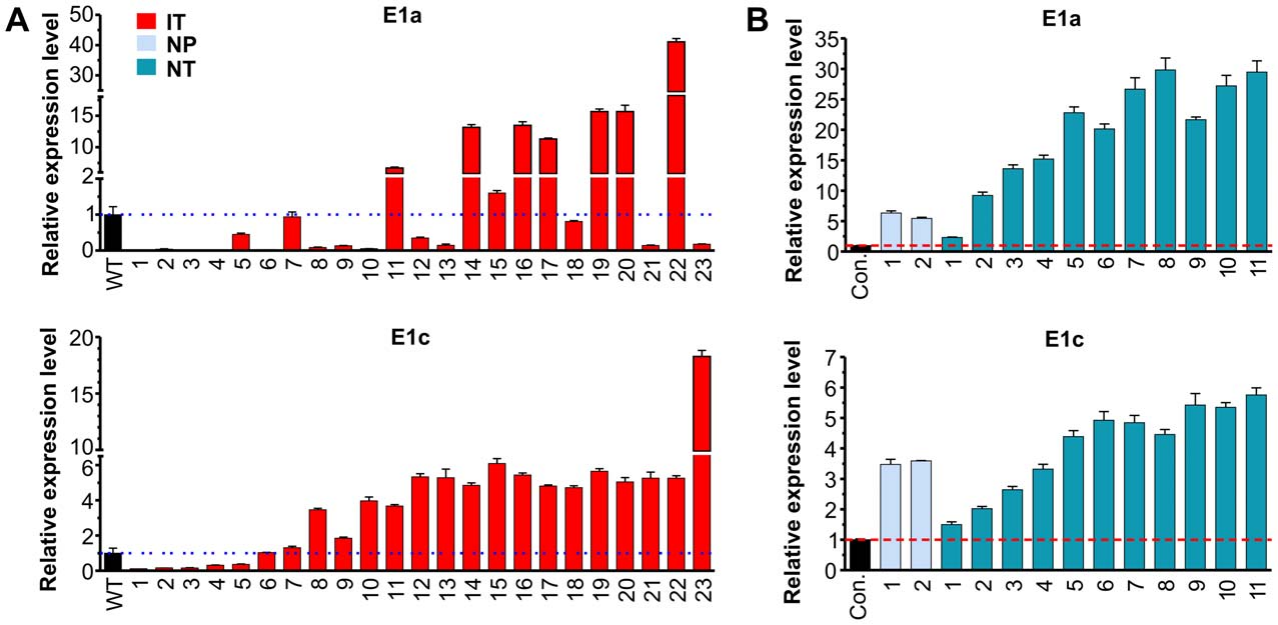
Examination of alternative promoter usage at the 5′ end of the Notch1 gene. Transcripts containing the alternative (E1a) and conventional (E1c) exon 1 were amplified using specific primers designed by Gomez-del Arco et al. The levels in each pre-tumor or tumor samples were compared to the average levels in wild type mice obtained from 6 FVB/N (A) and 4 C57BL/6 (B) mice, respectively.

A second mechanism has been suggested by the discovery of alternative promoters used to initiate transcription upstream of the conventional exon1 (E1c), thus creating an alternative exon 1, named E1a, which is then joined with exon 3 by RNA splicing [28]. This transcript codes for a product which can also be activated in a ligand-independent manner. Loss of Ikaros function is thought to correlate with this alternative promoter usage. Therefore, we determined the levels of Notch1 transcripts containing the E1a or E1c exon (Fig. 6). In Id1-expressing tumors, several samples clearly expressed 2-fold higher levels of the E1a transcript compared to the average level of wild type controls (Fig. 6A). Using the Pearson test (χ^2^), we detected a significant correlation between the levels of E1a and Notch1 transcripts in this group of samples. However, no correlation was found with the expression levels of other Notch target genes.

Consistently, the levels of the E1a transcript were significantly higher in all of the N1C-expressing tumor samples except for NT1 (Fig. 6B). In the pre-tumor samples, the levels of the E1a transcript were also very high. These observations raised the possibility that Notch signaling itself stimulates the transcription initiation at the alternative promoter and generates E1a-containing transcripts. Therefore, the increased levels of E1a transcripts in some of the Id1-expressing tumors could be due to high levels of Notch function in the tumors cells but expression of E1a transcripts could perpetually reinforce Notch signaling. However, it is noted that not all Id1-expressing tumors with robust Notch activities produce this transcript, suggesting that additional levels of control are at play.

### Point mutations accumulate in Id1 but not N1C-expressing tumors

**Figure 7:**
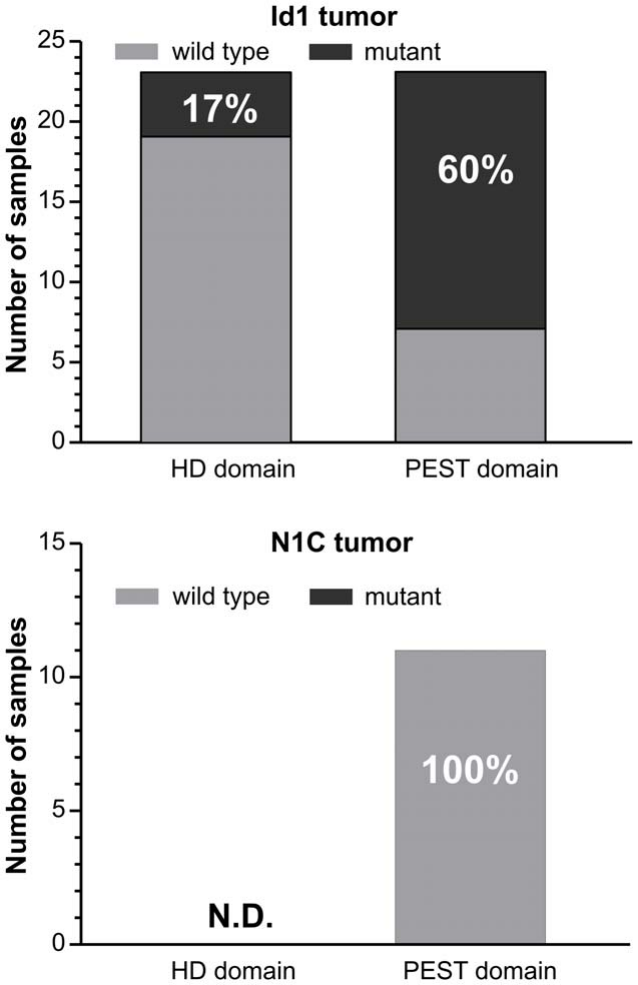
Examination of point mutations in Id1 and N1C-expressing tumors. The heterodimerization domain and the PEST domain of the Notch1 coding sequence were PCR amplified separately and the products were sequenced. Bar graphs show the numbers of tumors with wild type (grey) or mutant (black) genotypes. The percentage of each genotype is shown within the bars. The natures of the mutations are summarized in Fig. 10.

It is also well known that point mutations in the Notch1 gene result in mutant proteins with enhanced activities. Mutations in the heterodimerization domain (HD), which occur more frequently in human T-ALL, expose the protease cleavage sites and cause ligand-independent release of the intracellular domain [41]. On the other hand, missense or frame-shift mutations in the PEST domain, more commonly found in mouse T cell lymphomas, stabilize Notch1 proteins by preventing ubiquitin-mediated degradation. Sequence analyses of the HD and PEST regions in Notch1 transcripts expressed in Id1 tumor samples revealed such mutations at frequencies of 17% and 60% respectively (Fig. 7). In contrast, none of the N1C tumors harbor any mutations in the PEST region and it would be unlikely that they would have mutated HD domain which is known to be rare in mouse T cell tumors (Fig. 7). These results suggest that the gain-of-function mutations in the Notch1 gene provides tumor cells with selective advantage despite the presence of other Notch-activating mechanisms as described in previous sections. However, the N1C tumors have no need for these mutations as they already express high levels of a constitutively active form of Notch1.

### Id1 expression in normal thymocytes does not cause cryptic transcription initiation of the Notch1 gene

**Figure 8:**
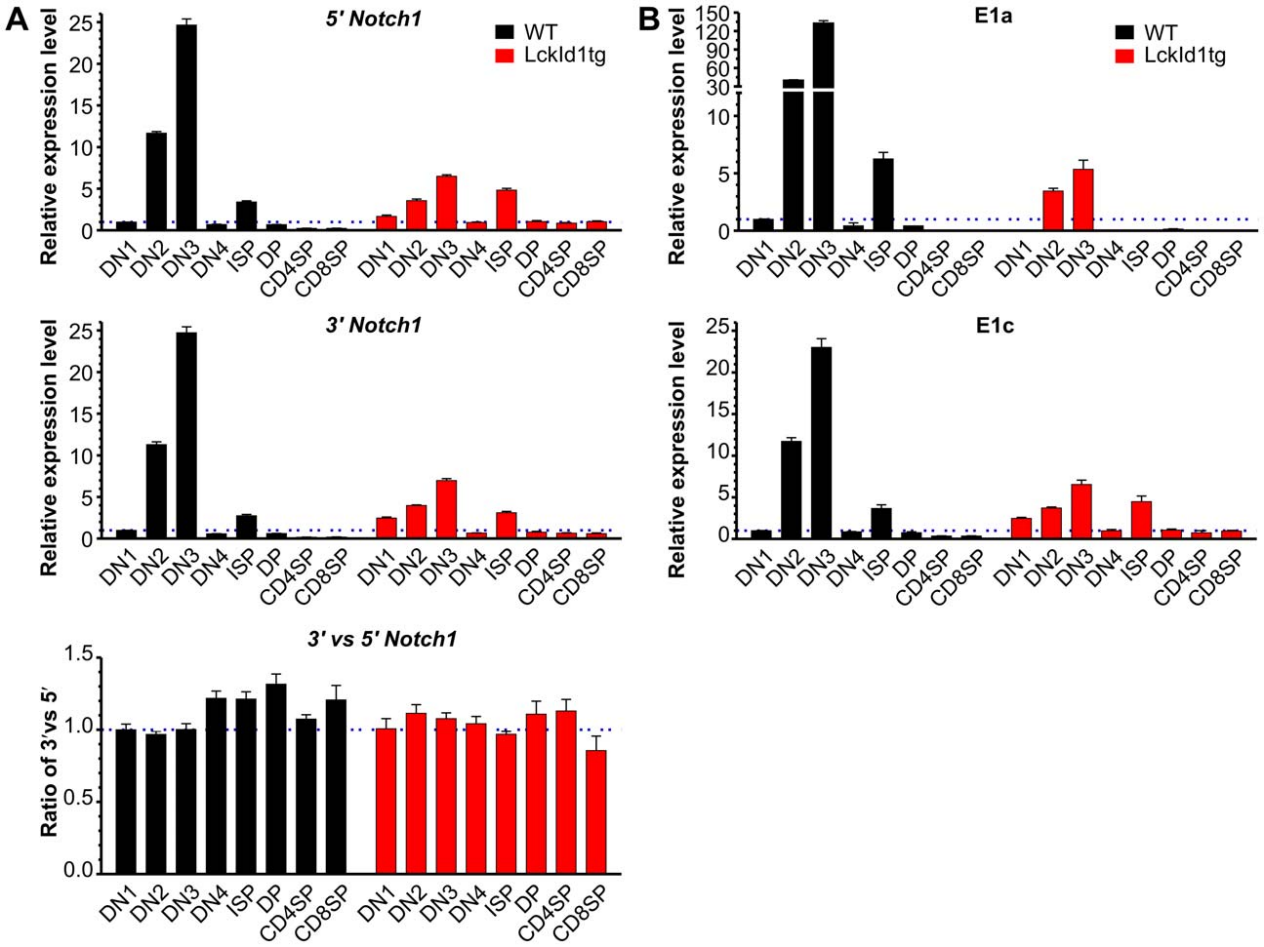
Analyses of cryptic transcriptional events in normal thymocytes. Thymocytes from one-month-old wild type and Id1 transgenic mice were sorted based on their developmental stages as described in Materials and Methods. Levels of indicated Notch1 transcripts were determined as described in Figures 5 and 6. Data are presented relative to the levels in wild type µg DN1 thymocytes.

Considering that 40% of the Id1 tumors exhibited intragenic initiation of transcription and 35% of them showed elevated expression of E1a containing transcripts, we were interested in determining if Id1 expression had any direct consequence in these cryptic transcriptional events. Wild type and pre-malignant Id1 transgenic thymocytes were fractionated by cell sorting based on their developmental profiles, namely CD4 and CD8 double negative (DN), double positive (DP) and single positive (SP) stages. Within the DN population, the cells were further divided into DN1 to DN4 subsets based on CD44 and CD25 surface phenotypes. Immature CD8 single positive cells, which represent cells at the DN to DP transition, were identified based on low levels of TCRβ expression. Consistent with previous observations, endogenous Notch1 expression detected by either 5′ or 3′ primer sets was the highest at DN2 and DN3 stages [42]. Id1 expression inhibited Notch1 expression which is expected as E2A proteins are thought to be activators of Notch1 transcription [37]. However, comparison between the levels of Notch1 detected by 3′ primer pair to those with 5′ primer pair did not reveal any discrepancy in either wild type or Id1 transgenic thymocytes (Fig. 8A), as detected in Id1 tumors. These results thus suggest that inhibition of E2A function by Id1 does not by itself induce intragenic transcription initiation.

Likewise, examination of E1a and E1c containing transcripts showed high levels of expression at DN2 and DN3 stages in wild type mice. Id1 transgenic mice produced much lower levels of both types of transcripts, suggesting that Id1 does not specifically initiate Notch1 transcription at the promoter near E1a (Fig. 8B). More significantly, E1a transcripts were more dramatically up-regulated at the DN2 and DN3 stages of wild type thymocytes than the E1c transcripts (e.g. 133 fold versus 23 fold in DN3 cells). These results may be interpreted to mean that transcription from the alternative promoter that gives rise to E1a-containing transcripts is a normal event which occurs as T cell progenitors develop in the thymus and at the stages when Notch function is critically required. Production of Notch1 proteins which can be activated in a ligand-independent manner would be able to better meet the demand for Notch function. Furthermore, it is interesting to note that Id1 expression had a more profound inhibitory effect on expression of the E1a transcript than the E1c transcript, which might provide a clue as to how Id1 effectively suppresses T cell differentiation [12], [42], [43].

### Reduction in Ikaros expression in Id1 tumors

**Figure 9:**
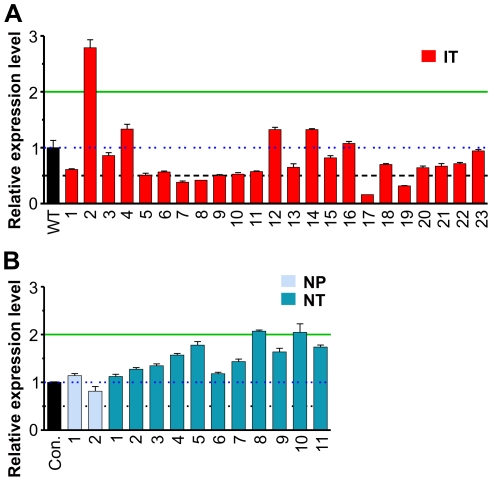
Levels of Ikaros expression. Real-time PCR analyses were performed using the same cDNA samples used for Figures 3–6. The levels of Ikaros in each pre-tumor or tumor samples were compared to the average levels in wild type mice obtained from 6 FVB/N (A) and 4 C57BL/6 (B) mice, respectively.

Since Ikaros is implicated in the suppression of these cryptic transcriptional events, we determined the levels of Ikaros in Id1-expressing tumors in comparison to those in N1C-expressing tumors [26], [28]. In 60% of Id1 tumors (18 samples), Ikaros expression was reduced to less than 70% of the wild type level (Fig. 9A). However, none of the N1C tumors exhibited decreased Ikaros expression and some had up to 2-fold increases (Fig. 9B). These data suggest that diminution of Ikaros expression offers selective advantage in Id1 tumor cell survival or growth, probably by allowing the usage of alternative promoters in the Notch1 locus. While the majority of the samples thought to produce intragenic (9 out of 9) and E1a (5 out of 7) transcripts indeed had reduced levels of Ikaros, there were a few exceptions, namely, two tumors with normal levels of Ikaros generated E1a transcripts and three tumors with low levels of Ikaros did not have these cryptic transcripts.

## Discussion

**Figure 10:**
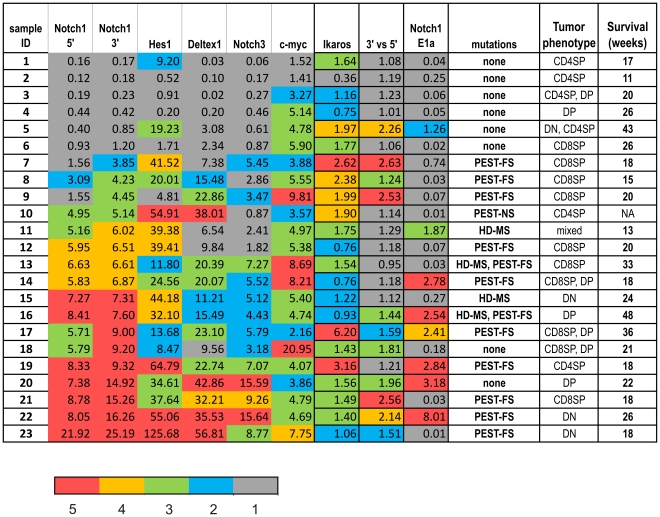
Summary of Notch activities and Notch activating mutations or alterations in Id1 tumors. Quantitative data from Figures 3, 5, 6 and 9 were color coded manually based on the value of each sample. Five color-coded brackets (red being the highest and gray the lowest as indicated by the legend) are used to represent the quantities of a given gene by dividing the second highest value with 5 and assigning the color for each sample according to its level of expression of the gene. The second highest value was used to better represent the all group by eliminating one outlier. Notch activities are defined by the collective levels of Notch1, Hes1, Deltex1, Notch3 and c-myc shown in color blocks without borders. Cryptic transcription of Notch1 was quantified by the level of E1a transcripts and the ratio between 3′ and 5′ Notch1 transcriptions. The color codes for the level of Ikaros were inverted such as red means the lowest and gray shows the highest. The nature of the point mutations in the HD and PEST domains of the Notch1 gene found in Id1 tumors were listed as FS (frame-shift), NS (nonsense) and MS (missense). The phenotypes of the tumors are shown as CD4 and CD8 double negative (DN), double positive (DP) and single positive (SP).

In this report, we have examined Notch activities in a series of T cell lymphomas developed in Id1 transgenic mice in comparison to those in tumors from ROSA26-N1C/lck-Cre mice. Several Notch-activating mechanisms including cryptic transcription initiation at the 5′ end or intragenic regions and point mutations have also been analyzed. By putting these pieces of information together as illustrated in Fig. 10, we were able to assess the contribution of aberrant Notch activation to T cell lymphomagenesis in the context of loss of E protein function. Furthermore, we were able to evaluate the contribution of different mechanisms that are thought to cause Notch activation to the overall Notch activity in tumor cells and a number of lessons have been learned.

First, while the majority of Id1 tumors showed evidence of Notch activation based on up-regulation of the expression of Notch1 and to a varying degree, its downstream targets, 26% of the tumors did not appear to have significantly elevated Notch activities. This suggests that other oncogenic factors beside Notch are able to cooperate with loss of E2A function in tumorigenesis. Second, although levels of Hes1, Deltex1 and Notch3 are generally in parallel with that of Notch1 except in a few cases, the level of c-myc did not correlate with Notch1 levels based on statistical analyses. The level of c-myc fluctuated within less than 10 folds compared to wild type control regardless the levels of Notch activity, suggesting that Notch-independent activation of the *c-myc* gene also occurs. One extreme example is sample IT18, which produced the highest level of c-myc but very low levels of Hes1, Deltex1 and Notch3.

With regard to the cryptic transcriptional events, their effects on the overall Notch activity in Id1 tumors are also variable. Intragenic transcription seemed to be associated with high Notch activities in samples IT20-22. However, it did not lead to high levels of Notch activities in IT5, 7 and 9. Alternative promoter usage leads to production of E1a-containing transcripts, which encodes the protein also capable of ligand-independent activation. Levels of this transcript appeared to be higher in a fraction of tumors that have more abundant Notch activities, namely IT14-23, which is consistent with the observation that ectopically or endogenously activating Notch signaling results in an elevation in the levels of this transcript (Fig. 6 and 8). However, not all Id1 tumors with high levels of Notch activities expressed the E1a-containing transcript, suggesting the existence of additional layers of control. On the other hand, most of the N1C tumors transcribed high levels of the E1a message. Combined with the observation that E1a expression parallels with Notch activity in normal thymocytes, it implies that Notch plays a role in stimulating the transcription from the alternative promoter.

Mutations in the Notch1 coding sequences are frequently found in Id1 tumors. Combining the incidences found in either HD or PEST regions or both, the mutation rate is 66% although the majority of the mutations occurred in the PEST domain. It has been shown that mutations in the HD domain have a stronger oncogenic effect than those in the PEST domain when the mutant proteins are expressed in bone marrow progenitors [25]. Specifically, a PEST domain mutant, N1ΔP, was not able to induce T-ALL by itself in recipient mice transplanted with N1ΔP-expressing bone marrow cells but cooperated with an activated *K-ras* oncogene in leukemogenesis. Therefore, these mutations may contribute to the overall Notch activity in Id1 tumors by stabilizing the proteins or potentiating activation by proteases [44], even though these mutations may be insufficient to induce T-ALL alone. In light of the recent discovery of cryptic transcription of the Notch1 gene, PEST domain mutations could synergize with cryptic transcripts to boost Notch activity. However, our data presented here could not ascertain if the mutation resides in the cryptic transcripts. Regardless, stabilization of Notch1 would add to the overall activity.

Comparison of the aberrant transcriptional events and mutations in the Notch1 sequence between Id1 and N1C-expressing tumors reveals that natural selection takes place for gain of Notch function during tumorigenesis in Id1 transgenic mice. In contrast, such a selection is not necessary for N1C-expressing tumors as Notch signaling is already extremely high in the mice, which develop lymphoma at a much higher rate. Although N1C tumors produce abundant E1a-containing transcripts, N1C tumors do not exhibit any intragenic transcriptional initiation. Likewise, no PEST domain mutations were found in the coding sequences of these tumors. Consistent with this notion, varying levels of Notch receptor expression and Notch activities were detected in different Id1 tumors. Judging from the levels of Notch target gene expression, some Id1 tumors have Notch activities as high as the N1C-expressing tumors whereas others have minimal Notch function. It is also worth noting that expression levels of different Notch target genes are not uniform in each Id1 tumor or among all samples. Although Id1 transgenic mice consistently develop large thymic lymphomas, the addiction to diverse aspects of Notch activity varied significantly depending on the existence of other genetic or epigenetic alterations in each tumor. This is consistent with our previous finding that expression of Hes1 was able to dramatically potentiate tumorigenesis in Id1 transgenic mice [15].

Our study shows that Notch activities are clearly advantageous for lymphomagenesis in Id1 transgenic mice, probably through enhanced survival and/or proliferation of tumor cells. However, the onset of T cell lymphoma or tumor-free survival of Id1 transgenic mice does not appear to be proportional to the levels of Notch activities or depend on Notch activation. As shown in Fig. 10, sample IT1-4 had no mutations in the Notch1 gene or aberrant Notch1 transcription. Yet, mice with these tumors succumb to T cell lymphoma faster than the 4 mice with the highest Notch activities in their tumors (IT20-23). The same also holds true for the tumors in Group B, which had a wide range of survival rate. This suggests that inhibition of E2A function by Id1 remains to be instrumental for T cell lymphomagenesis. The fact that 21 out of 23 tumors maintained very high levels of transgene expression in Id1 tumors supports such a notion. Furthermore, two separate studies showed that about 25% of TAL1-expressing tumors also lack Notch1 mutations, suggesting that inhibition of E proteins remains relevant [38], [39]. It has been postulated that pre-TCR signaling is necessary for T cell lymphomagenesis in TAL1 and/or LMO1 induced oncogenesis [45]. Our previous studies have provided evidence to suggest that Id1 potentiate pre-TCR signaling during T cell development [13], [43], [46]. Alternative possibilities may be that the developmental defects caused by E2A deficiency arrest cells at certain stages when the cells are particularly vulnerable to oncogenic stimulation through a variety of secondary mutations and that E proteins could also be important for expression of tumor suppressors. Therefore, Notch activation is undoubtedly an important factor but is unlikely the only factor. When considering therapeutic strategies, it is thus not sufficient to simply target Notch signaling pathways as they are not the sole initiating force for T-ALL. Clinical studies already indicate that T-ALL patients harboring Notch mutations have more favorable prognosis and Notch-target therapy has complications [21], [32], thus underscoring the necessity to look not only within but also beyond Notch pathways.

## Materials and Methods

### Mouse models, tumor tissue collection and characterization

Mice were housed in the Laboratory Animal Resource Center of the Oklahoma Medical Research Foundation in a specific pathogen free environment. This project was approved under the protocol S0147-3 by the Oklahoma Medical Research Foundation Institutional Animal Care and Use Committee.

Id1 transgenic mice on the FVB/N background were previously described [12]. To generate ROSA26-N1C mice, the construct was created by inserting a SalI-SacII fragment containing human N1C and GFP sequences isolated from a retroviral construct into the pBigT vector [16], [47], from which a PacI-AscI (partial digest) fragment was isolated and inserted into the ROSA26PA vector [48]. The construct was linearized and electroporated into 129X1/SvJ embryonic stem cells [49]. ES cells containing properly targeted ROSA26-stop-N1C alleles were identified by Southern blotting and PCR assays and then injected into C57BL/6 blastocysts. ROSA26-stop-N1C progenies were backcrossed to C57BL/6 mice for 10 generations. ROSA26-N1C mice were then crossed with lck-Cre transgenic mice (Jackson laboratory). Lymphoma development was monitored at least twice a week. Animals showing symptoms of tumor formation include hunch back, ruffled fur and labored breathing were sacrificed for tissue collection and analyses. Single cell suspensions were used for flow cytometry analysis and solid tumor tissues were frozen for isolation of total RNA later.

### Flow Cytometry and cell sorting

Single cell suspensions from tumor tissues were stained with fluorescent-conjugated antibodies against CD4, CD8 TCRβ and CD25, and analyzed on a BD LSRII flow cytometer using standard procedures. To delineate DN thymocyte populations, thymocyte suspensions were stained with lineage specific antibodies for CD8, TCRγ/δ, B220, Gr1 and CD3. Upon gating on lineage negative cells, DN populations were further assayed based on CD44 and CD25 staining. The CD8 immature single positive (ISP), DP, and CD4 as well as CD8 single positive (CD4SP and CD8SP) cell populations were isolated based on CD4, CD8 and TCRβ expression after gating out all cells staining with propidium iodide. The ISP population was defined as CD4^−^CD8^+^TCRβ^lo^ cells. Cell sorting was performed on a MoFlo (Dako Colorado, Inc., Fort Collins, CO) using thymocytes from mice of different genotypes at 3 to 5 weeks of age.

### Real time PCR and RT-PCR analyses

Total RNA from sorted T cells and tumor tissues was isolated using Trizol reagent per manufacturer's protocol. Total RNA was then used to synthesize cDNA using M-MLV reverse transcriptase (Invitrogen, Carlsbad, CA). Quantitative PCR was performed using Power SYBR Green PCR Master Mix (Applied Biosystem, Foster City, CA) on an Applied Biosystems 7500 Real Time PCR and software analysis system. Levels of transcripts were normalized against that of β-actin by calculating ΔC_T_. Expression levels relative to that of the reference sample were determined by using the 2^−ΔΔCT^ formula. The primers used are β actin F, GGCTGTATTCCCCTCCATCG, β actin R, CCAGTTGGTAACAATGCCATGT; Notch1 5′ F, TGTGGACGGCGTGAATACCT, Notch1 5′ R, GGGCATGAGCTGACATTCGT; Notch1 3′ F, CTGAAGAACGGAGCCAACAA, Notch1 3′ R, CAGCAACACTTTGGCAGTCTCA; Notch1 E1a F, GACCCCATTGCTCTCCTTGG , Notch1 E1a R, TGATGGACCTGGAAGGGAAGA; Notch1 E1c F, AATGGAGGGAGGTGCGAAGT, Notch1 E1c R, GATTGGAGTCCTGGCATCGT; Hes1 F, CCAGCCAGTGTCAACACGA, Hes1 R, AATGCCGGGAGCTATCTTTCT; c-myc F, CACCACCAGCAGCGACTCT, c-myc R, TCCACAGACACCACATCAATTTCT; Deltex1 F, AGGATGTGGTTCGGAGGTACAT, Deltex1 R, CGCTCCATGCAAATGGTACA; Notch3 F, ATGCCCAGGGTGTCTTCCA, Notch3 R, GCAGTAGAGCCATCTGCCATTC; total Id1 F, AGGTGAACGTCCTGCTCTAC, total Id1 R, GTCCCGACTTCAGACTCCG; human Notch1 F, TGTCTGCCGACGCACAA, human Notch1 R, CGTCGTGCCATCATGCAT; Ikzf1 F, AGGGTCAAGACATGTCCCAAGT, and Ikzf1 R, CCCCTTCATCTGGAGTGTCACT.

For detection of exon 27b-containing transcript, RT-PCR was carried out under the following conditions: 94°C for 3 minutes, followed by 38 cycles of 94°C for 30 seconds, 57°C for 30 seconds and 72°C for 45 seconds, and a final step of 72°C for 7 minutes. The primers are exon 27b, CTCTTCAGGGATGGGTTCAT and exon 33, TGTTCTGCATGTCCTTGTTG as previously described [26]. PCR products of β-actin, which served as cDNA quality controls, were generated with the following primers, GATGGTGGGAATGGGTCAGAAGGACTC, and GAGGTCTTTACGGATGTCAACGT CACA.

### Sequence analysis of the Notch1 allele

The HD domain and PEST domain of the Notch1 gene were amplified from cDNA synthesized from individual tumors with the following primers: GTGATGGCCACTGCGACCAGGGCTGTAAC and CGACTGAGTCCTCGCCGAGGGGCTCT, GTGGCTTCCCCGGCTCCAGAATG and GAAGCTGGGGTCCTGCATCCCACATC. The resulting PCR products were purified with DNA Gel Extraction Kit (Millipore, Billerica, MA), and then were analyzed by DNA sequencing.

### Statistical analysis

The statistical significance of the difference in gene expression levels between different sample groups was judged by unpaired *t* test. The correlation between different genes expressed in the same sample was determined by performing Pearson tests using the Prism 5 software.
